# Métastase parotidienne d’un cancer du sein: à propos d’un cas et revue de la littérature

**DOI:** 10.11604/pamj.2017.27.79.8876

**Published:** 2017-06-02

**Authors:** Fatimazahra El M’rabet, Rajae Kanab, Taoufiq Ameuraoui, Fatoumata Sidibe, Meryem Azegrare, Samia Arifi, Moustapha El Maaroufi, Afaf Amarti, Nawfal Mellas

**Affiliations:** 1 Service d’Oncologie Médicale, CHU Hassan II Fès, Maroc; 2 Service de Radiologie, CHU Hassan II Fès, Maroc; 3 Laboratoire d’Anatomopathologie, AL Azhar Fès, Maroc

**Keywords:** Breast cancer, parotid gland, distant metastasis, Cancer du sein, glande parotide, métastase à distance

## Abstract

La localisation au niveau de la parotide des métastases du cancer du sein est très rare, quelques cas ont été rapportés dans la littérature. On rapporte le cas d'une patiente de 43 ans, qui a été traitée pour un cancer du sein droit, et qui a présenté deux ans après la fin du traitement une métastase au niveau de la parotide gauche, confirmée histologiquement.

## Abstract (en)

Parotid metastasis from carcinoma of the breast is very rare, only a few cases have been reported in the literature. We here report the case of a 43-year old patient treated for right breast cancer in whom left parotid metastasis (confirmed histologically) occurred two years after the end of the treatment.

## Introduction

Les tumeurs des glandes salivaires sont des tumeurs rares, et particulièrement les métastases [1]. La glande parotide reste une localisation exceptionnelle d'une métastase d'un cancer du sein. On rapporte un cas de métastase parotidienne d'un carcinome canalaire infiltrant du sein déjà traité avec une revue de la littérature.

## Patient et observation

**Figure 1:**
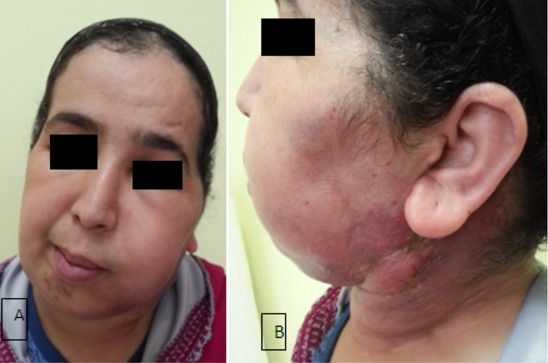
Tuméfaction en regard de la loge parotidienne avec une paralysie faciale droite

**Figure 2:**
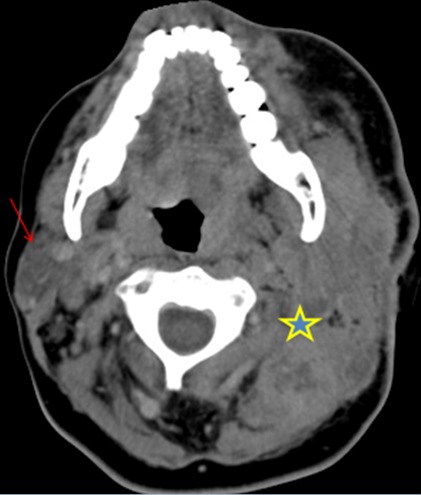
Coupes axiales tomodensitomériques (TDM): processus lésionnel tumoral centré sur la région parotidienne gauche (étoile) largement étendu aux structures musculaires latérocervicales homolatérales, avec infiltration de la peau et de la graisse parapharyngée: absence d’anomalie de la parotide droite (flèche)

**Figure 3:**
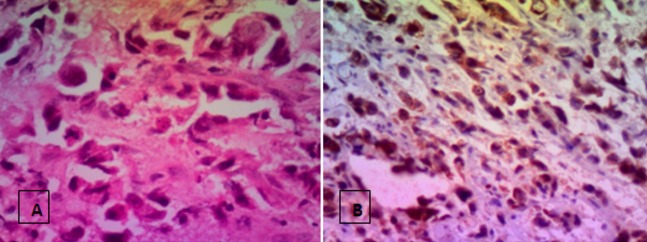
A) métastase d’un adénocarcinome bien différencié HE*200; B) expression de le CK 7 par les cellules tumorales HE*200

Les auteurs déclarent avoir pris le consentement de la patiente pour publier ses renseignements cliniques et sa photo. Mme H.B, âgée de 43 ans, mariée et mère de 3 enfants, a comme facteur de risque le cancer du sein dû à la contraception orale depuis 8 ans. Suivie depuis 2012 pour un cancer inflammatoire du sein droit, tumeur classée initialement T4d N1 M0 avec à l'immunohistochimie une forte expression des récepteurs hormonaux et absence d'amplification du gène HER 2 neu, elle a reçu une chimiothérapie néo adjuvante (schéma séquentiel à base d'anthracyclines, puis les taxanes) avec une bonne réponse clinique et biologique à la chimiothérapie, puis a été opérée: mastectomie droite avec curage ganglionnaire axillaire (chirurgie type patey), une radiothérapie sur la paroi et a été mise sous hormonothérapie adjuvante type tamoxifène depuis le mois d'aout 2013. Apres deux ans d'hormonothérapie la patiente a présenté une tuméfaction de la loge parotidienne (Figure 1), avec paralysie faciale périphérique (Figure 1), ce qui a motivé la patiente a consulté chez un ORL ou elle a eu un scanner cervical (Figure 2) montrant un processus lésionnel tumoral centré sur la région parotidienne gauche (étoile) largement étendu aux structures musculaires latérocervicales homolatérales, avec infiltration de la peau et de la graisse para pharyngée, puis une biopsie de la masse parotidienne dont l'histologie est revenue en faveur d'une métastase parotidienne, d'un adénocarcinome d'origine mammaire: cytokératine positive (CK 7), absence d'expression des récepteurs hormonaux, et absence d'amplification du gène HER 2 neu (Figure 3). Le bilan d'extension n'a pas révélé la présence d'autres localisations secondaires ou de récidive locale de la tumeur. La patiente a été mise sous chimiothérapie type paclitaxel 175mg/m2 chaque trois semaines pendant 3 cycles.

## Discussion

Les métastases au niveau des glandes salivaires sont des entités très rare, qui ne touchent, dans ces glandes, que la glande parotide et les glandes sous mandibulaires. Elles ne dépassent pas 10% des tumeurs malignes touchant les glandes salivaires [2]. La physiopathologie de ces métastases peut être due à 3 voies de dissémination tumorale: envahissement locorégionale direct par la tumeur primitive; par voie lymphatique pour les tumeurs ORL (carcinome épidermoide cutanée, tumeur thyroïdienne); par voie hématogène pour les tumeurs à distance, les tumeurs les plus fréquemment rencontrées sont: le poumon, le rein et le sein [3,  4].

Cliniquement une tumeur de la parotide peut se manifester par [5]: présence d'une masse ferme et décelable lors de la palpation généralement situé sous l'oreille et indolore; douleurs au niveau du visage, picotement, gênes; anomalies dans la motricité des muscles du visage; difficulté à avaler; paralysie faciale [6]. L a présence de métastase parotidienne rend la tumeur de mauvais pronostic selon les petites séries et les cas rapportés dans la littérature [7,  8]. La chirurgie reste le seul traitement qui peut améliorer le pronostic de ces tumeurs suivie de radiothérapie locale [9]. Le traitement médicale qui peut être proposé dépend du type histologique et de l'immuno histochimie de la récidive tumorale, dans notre cas, la patiente avait des récepteurs hormonaux initialement positifs, mais la métastase parotidienne était triple négatif (ce qui est possible après un traitement hormonal) avec une tumeur très localement avancée inopérable d'où la décision de la mettre sous chimiothérapie. L'étude histologique a un grand intérêt pour poser le diagnostic et éliminer un primitif parotidien. Le carcinome du canal salivaire peut composer de cellules: solide, papillaire, kystiques et cribriformes, et il ressemble ainsi au carcinome canalaire d'origine mammaire [10]. Les résultats de l'examen clinique et les antécédents des patients peuvent aider à poser le diagnostic. Pour les tumeurs qui n'expriment pas les récepteurs ostrogéniques et qui expriment l'ACE le diagnostic de métastase de cancer mammaire reste peu probable par rapport au carcinome du canal salivaire [11,  12]. Dans certains cas publiés dans la littérature l'immuno histochimie n'a pas été faite et le diagnostic a été retenu sur des arguments cliniques [13].

## Conclusion

Les métastases parotidiennes proviennent essentiellement des tumeurs ORL. Dans 10 à 20% seulement des cas, ces métastases proviennent d'autres localisations. Elles peuvent être soit révélatrices de la tumeur primitive ou survenir quelques années après un traitement curatif de la tumeur primitive. L'histologie et l'immunohistohichimie vont permettre de poser le diagnostic de métastase et de préciser l'origine de la tumeur primitive.

## Conflits d’intérêts

Les auteurs ne déclarent aucun conflit d'intérêt.
